# Automatic Continuous Control of Cuff Pressure and Subglottic Secretion Suction Used Together to Prevent Pneumonia in Ventilated Patients—A Retrospective and Prospective Cohort Study

**DOI:** 10.3390/jcm10214952

**Published:** 2021-10-26

**Authors:** Lucyna Tomaszek, Jarosław Pawlik, Henryk Mazurek, Wioletta Mędrzycka-Dąbrowska

**Affiliations:** 1 Faculty of Medicine and Health Sciences, Andrzej Frycz Modrzewski Krakow University, 30-705 Kraków, Poland; ltomaszek@afm.edu.pl (L.T.); jarek.pawlik@yahoo.com (J.P.); 2 Department of Pneumonology and Cystic Fibrosis, National Institute of Tuberculosis and Lung Disorders, 34-700 Rabka-Zdrój, Poland; hmazurek@igrabka.edu.pl; 3 Health Institute, State University of Applied Sciences in Nowy Sącz, 33-300 Nowy Sącz, Poland; 4 Department of Anesthesiology Nursing & Intensive Care, Faculty of Health Sciences, Medical University of Gdansk, 80-211 Gdańsk, Poland

**Keywords:** patient care bundles, taper-shaped cuff, subglottic secretion suction, continuous control pressure

## Abstract

The ventilator bundle consists of multiple methods to reduce ventilator-associated pneumonia (VAP) rates in Intensive Care Units (ICU). The aim of the study was to evaluate how the continuous automatic pressure control in tapered cuffs of endotracheal/tracheostomy tubes applied along with continuous automatic subglottic secretion suction affect the incidence of VAP. In the prospective cohort (*n* = 198), the standard VAP bundle was modified by continuous automatic pressure control in taper-shaped cuff of endotracheal/tracheostomy tubes and subglottic secretion suction. VAP incidence, time to VAP onset, invasive mechanical ventilation days/free days, length of ICU stay, ICU mortality, and multidrug-resistant bacteria were assessed and compared to the retrospective cohort (*n* = 173) with the standard bundle (intermittent cuff pressure of standard cuff, lack of subglottic secretion suction). A smaller incidence of VAP (9.6% vs. 19.1%) and early onset VAP (1.5% vs. 8.1%) was found in the prospective compared to the retrospective cohort (*p* < 0.01). Patients in the prospective cohort were less likely to develop VAP (RR = 0.50; 95% CI: 0.29 to 0.85) and early-onset VAP (RR = 0.19; 95% CI: 0.05 to 0.64) and had longer time to onset VAP (median 9 vs. 5 days; *p* = 0.03). There was no significant difference (*p* > 0.05) between both cohorts in terms of invasive mechanical ventilation days/free days, length of ICU stay, ICU mortality and multidrug-resistant bacteria. Modification of the bundle for prevention of VAP can reduce early-onset VAP and total incidence of VAP and delay the time of VAP occurrence.

## 1. Introduction

In a large number of cases, therapy in Intensive Care Units (ICU) is associated with opening the airways with an endotracheal or tracheostomy tube and supporting breathing with a ventilator [1], which promotes lower respiratory tract infections. Ventilator-associated pneumonia (VAP) is one of the most serious types of these infections. It is defined as symptoms of pneumonia occurring 48 h after the onset of artificial airway and mechanical ventilation [2,3].

The prevalence of VAP depends on the diagnostic criteria used [4], the geographic region, and the type of hospital [5,6]; this ranges in adult patients from 4% to 42%. According to a systematic review and meta-analysis conducted by Bonell et al. [7], which included 538,600 patients from 14 Asian countries, the incidence density of VAP/1000 ventilator days ranged from 9 to 18.5. In Poland, the incidence of VAP, according to data collected in 2013–2017, was 8–10%, and the incidence density of VAP per 1000 ventilator days was 12.3–13.7 [8,9]. VAP often had a high mortality rate in critically ill patients varying from 16.2% to 74.1% [10].

Artificial airway (endotracheal tube, tracheostomy tube) is one of the most important risk factors for VAP. It disrupts natural defense mechanisms (e.g., cough, laryngeal reflex) and is a mechanical obstacle preventing effective mucociliary clearance. Moreover, it facilitates aspiration of contaminated gastric and oropharyngeal secretions into the bronchial tree: the secretion accumulating in the subglottic area passes through microchannels between the endotracheal/tracheostomy tube sealing cuff, and the tracheal wall to the bronchial tree [11].

VAP is a major therapeutic problem as it extends the duration of mechanical ventilation, and thus hospitalization, increasing its cost [9,12]. The implementation of the ventilator bundle in ICU patients may contribute to the reduction of VAP [13]. The most frequently proposed strategies include elevation of the head of bed to 30°–45°, daily “sedation vacation”, and selective oral decontamination [14]. As part of the VAP prevention bundle, Álvarez Lerma et al. [15] also recommended the use of measures to reduce the risk of micro-aspiration of secretions around the artificial airway cuff, such as cuff pressure control and subglottic secretion suction (SSS). Continuous pressure control might be beneficial thanks to the reduction of episodes of hypotension in the cuff, which can facilitate the passage of bacteria between the cuff and the tracheal wall. Reduction of VAP risk by continuous control of pressure was confirmed by Nseir et al. [16], although there was no impact on duration of mechanical ventilation, ICU length of stay, or mortality. It also appears that subglottic secretion suction may contribute to the reduction not only of the total incidence of VAP, but also of early VAP [17] and mortality [18]. The authors of in vitro experimental studies [19,20] suggested that the shape of the artificial airway cuff may also influence the amount of micro-aspiration content. However, a meta-analysis of randomized clinical trials conducted by Huang et al. [21] did not confirm the advantage of the tapered-cuff endotracheal tube over the standard-cuff endotracheal tube in reducing VAP and ICU mortality.

Given the above, we can conclude that the effect of the tapered-cuff endotracheal tube with the concomitant use of SSS and continuous cuff pressure control remains unexplored [22]. The aim of the study was to evaluate how the continuous automatic pressure control in tapered cuffs of endotracheal/tracheostomy tubes applied along with continuous automatic subglottic secretion suction affect the incidence of VAP.

## 2. Materials and Methods

### 2.1. Study Design, Setting

The study was conducted in a prospective cohort (intervention, study period from 1 June 2018 to 1 July 2019) and compared to the historical cohort (retrospective, from 1 May 2017 to 30 April 2018). The study was conducted in the Clinical Department of Anesthesiology and Intensive Therapy of the St. Raphael Hospital in Krakow at the University Department of Anesthesiology, Intensive Therapy and Emergency Medicine of the Faculty of Medicine and Health Sciences of the Andrzej Frycz Modrzewski Krakow University. The study was approved by the Bioethical Committee of the Andrzej Frycz Modrzewski Krakow University (opinion no. KBKA/34/O/2018). Written consent from the subjects or their surrogates was not required by the institutional review board as the VAP prevention bundle was one of the basic components of therapy in saving lives. The study was registered on Clinicaltrails.gov under number NCT04038814, https://clinicaltrials.gov/ct2/show/NCT04038814 (accessed on 9 September 2021). The study was conducted in accordance with the guidelines of the Declaration of Helsinki and STROBE (Strengthening the Reporting of Observational Studies in Epidemiology) [23].

### 2.2. Participants

The presence of an artificial airway and the duration of mechanical ventilation over 48 h were the conditions for qualifying patients for both cohorts. In the prospective cohort, patients admitted in ICUs already intubated (shorter than 12 h) with a tube other than a tube with a tapered cuff and channel for subglottic secretion suction, were re-intubated. Blue Rhino Ciaglia 2 percutaneous tracheostomy was performed under bronchofiberoscopy and ultrasound guidance between the 10th and 14th day after intubation in all patients with the expected need to maintain airway patency for more than 14 days.

Patients were excluded from the study if they were younger than 18 years, pregnant, nasally intubated or with tracheostomy upon ICU admission, intubated for longer than 12 h before ICU admission, or were previously enrolled in this study.

### 2.3. VAP Bundle

The basic components of the VAP prevention bundle included:

▪ basic rules of asepsis and hygiene of staff hands,
▪ raising the head of the bed at an angle of 30–45 degrees (implemented already upon admission to the ICU and verified every 4 h),
▪ limiting the use of sedation,
▪ moderately lung-sparing ventilation (VT 5–8 mL/kg due body weight, PEEP > 3 cm H_2_O with ventilator settings allowing for normocapnia and plateau pressure < 25 cm H_2_O) with rapid weaning from mechanical ventilation,
▪ closed system for suctioning secretions from the respiratory tract, replaced every 7 days or in the event of leakage,
▪ oral care with the use of 0.2% chlorhexidine digluconate solution every 12 h,
▪ the use of proton pump inhibitors with the gradual dose reduction and discontinuation of the drugs in the absence of risk factors for peptic ulcer disease,
▪ the use of anticoagulants.

Endotracheal tubes (Mallinckrodt™ TaperGuard Evac Oral Tracheal Tube, Covidien, Mansfield, MA, USA) or tracheostomy tubes (Shiley EVAC Tracheostomy Tube Cuffed Seal Guard, Covidien, Guardfield, MA, USA) with a tapered cuff and channel for subglottic secretion suction were used in all patients from the prospective cohort. The cuff pressure of the tubes was continuously measured automatically (Shiley Pressure Control, VBM Medizintechnik GmbH, Covidien, Germany) and was maintained at 25–30 mmHg. Continuous subglottic secretion suction above the cuff sealing the tube was performed with the Hersill Vacusill 3 Continuous-Intermittent apparatus (Madrid, Spain), with a designated suction force of 15–20 kPa (100–150 mm Hg or 136–204 cm H_2_O) by Evac suction tubing through the suction tube (DAR™, Italy) ID 3.5 mm.

Standard endotracheal tubes (Hi-Contour Oral/Nasal Tracheal Tube Cuffed Murphy Eye, Covidien, Mexico) or tracheostomy tubes (Shiley™ Single Cannula Tracheostomy Tubes, Covidien, Ireland) without a lumen for SSS were used in the retrospective cohort. The pressure in the cuff of the tubes was measured with a manual manometer (VBM Cuff Pressure Measuring, Germany) every 12 h or whenever hypotension or hypertension in the cuff was suspected (standard: 20–30 mm Hg).

### 2.4. Variables and Measures

For the purposes of this analysis, the following demographic and clinical data were collected for both cohorts: sex, age, body mass index, reason for hospitalization, comorbidities, APACHE 2 score, type of artificial airway, procalcitonin concentration, early VAP (diagnosed during the first 4 days of mechanical ventilation) and late VAP incidence (VAP diagnosed within or after the 5th day of mechanical ventilation) [3], time to VAP, number of days of mechanical ventilation, type of pathogen grown in the secretions of the lower respiratory tract, and number of deaths.

The APACHE II scale [24] was used to assess the severity of the patients’ health condition. The score ranges from 0 to 71 points. The higher the result, the worse the prognosis.

Diagnosis of VAP was suspected in patients receiving mechanical ventilation via chest echocardiography and the procalcitonin pulmonary infection score. Obtaining at least 6 points indicated the presence of VAP (Table 1) [25]. Additionally, chest radiographs (X-rays or computed tomography) were performed in all patients on admission to the ICU and when the ultrasound image suggested pneumonia. Chest radiographs were assessed and described by hospital radiologists. The assessment according to the CEPPIS criteria was performed by the anesthesiologist on duty.

### 2.5. Outcomes

The primary end point of the study was any incidence of VAP. Secondary outcomes included time to VAP onset, invasive mechanical ventilation days/free days, length of ICU stay, and mortality.

### 2.6. Statistics

At the stage of planning the prospective study, the minimum sample size was estimated based on the number of patients (N = 173) and the percentage of cases with VAP (*n* = 33; 19.07%) in the retrospective cohort. Assuming a decline in the percentage of patients with VAP up to 9% in the prospective cohort, with a 5% confidence level and power of a test of 80%, the calculations showed that the minimum size in the prospective cohort was 197 patients.

Continuous quantitative variables were characterized as medians and quartiles, while qualitative variables were presented as numbers of individual categories with the corresponding percentages. Because of the lack of a normal distribution of the variables in both cohorts, quantitative variables were compared between the prospective and retrospective cohorts using the Mann-Whitney U test. The distribution of variables was tested with the Shapiro-Wilk test. The chi-square test was applied to compare qualitative variables between the cohorts. The number of VAP cases between the prospective and retrospective cohorts was compared using one-way logistic regression (the regression coefficient and 95% confidence interval were determined). Furthermore, relative risk (RR) values and 95% confidence interval values for the probability of the occurrence of VAP were calculated using a relative risk calculator. The probability of remaining VAP free was demonstrated by the Kaplan-Meier method, and the two cohorts were compared using the log-rank test. The time to the development of VAP was censored for death. The incidence density of VAP was calculated by dividing the number of VAP cases by the number of ventilator days and multiplying by 1000. The incidence of VAP per 100 days of mechanical ventilation was also calculated and compared between the prospective and retrospective cohorts using the mid-*p* exact method available in the OpenEpi epidemiological calculator.

*p* values below 5% were considered statistically significant for the two-tailed tests. Statistical calculations were performed using STATISTICA v.13 (TIBCO Software Inc., Kraków, Poland (2017)).

## 3. Results

### 3.1. Demographic and Clinical Characteristics of the Groups

**Figure 1:**
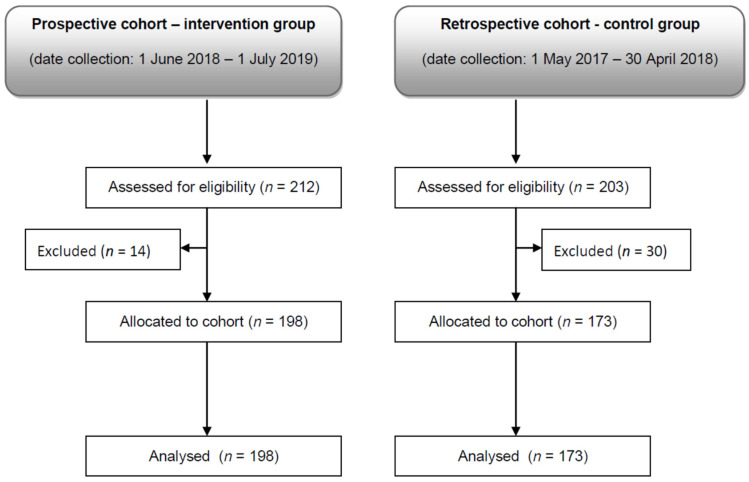
Flow diagram.

Initially, 415 patients were assigned to both groups. Of those, 44 (10.6%) patients were excluded due to failing to meet the eligibility criteria, i.e., 30 patients from the retrospective cohort, and 14 patients from the prospective cohort (Figure 1).

There were no significant differences between the prospective (*n* = 198) and retrospective (*n* = 173) cohorts in terms of age, sex, body mass index, APACHE-II score, chronic heart failure, chronic renal failure, chronic obstructive pulmonary disease, gastric and duodenal ulcer, diabetes mellitus, immunosuppression, smoking status, septic shock, tracheotomy, enteral nutrition, and antibiotics prior to VAP. The cohorts differed only in the cause of admission to the ICU and the number of patients diagnosed with hepatic failure. In the prospective cohort there were more hospitalizations for general surgical reasons (*p* = 0.004) and chronic liver failure (*p* = 0.03) than in the retrospective cohort. The detailed demographic and clinical characteristics of the patients are presented in Table 2.

Infection of other than the respiratory system was reported in every third ICU patient. Statistical analysis showed no differences (*p* > 0.05) between the prospective and retrospective cohort in the incidence of co-infections, such as: urinary tract infections (*n* = 30; 15.2% vs. *n* = 26; 15.0%), blood infections (*n* = 23; 11.6% vs. *n* = 22; 12.7%), infections of the peritoneal cavity and gastrointestinal tract (*n* = 27; 13.6% vs. *n* = 15; 8.7%), surgical wound infections (*n* = 11; 5.6% vs. *n* = 8; 4.6%) and central nervous system infections (*n* = 6; 3.0% vs. *n* = 5; 2.9%).

### 3.2. Prevalence of VAP

A smaller incidence of VAP (*n* = 19; 9.6% vs. *n* = 33; 19.1%; *p* < 0.01) and early-onset VAP (*n* = 3; 1.5% vs. *n* = 14; 8.1%; *p* = 0.0026) was found in the prospective compared to the retrospective cohort. The totals of late-onset VAP were similar in both cohorts (*n* = 16; 8.1% vs. *n* = 19; 11%; *p* = 0.34). The incidence density of VAP per 1000 ventilator days was 7.8 and 11.2.

Univariate logistic regression analysis showed that the continuous control of endotracheal/tracheostomy tube cuff pressure together with continuous subglottic secretion drainage were protective factors against VAP (OR = 0.45; Cl 95%: 0.25 to 0.83) and early-onset VAP (OR = 0.17; Cl: 0.05 to 0.60). Patients in the prospective cohort were 81% less likely to develop early-onset VAP (RR = 0.19; 95% CI: 0.05 to 0.64; *p* = 0.0076) and were 50% less likely to develop total VAP than in the retrospective cohort (RR = 0.50; 95% CI: 0.29 to 0.85; *p* = 0.01).

### 3.3. Time to Onset of VAP and the Probability of Remaining VAP-Free

In the prospective cohort, the median time from intubation to VAP was significantly longer than in the retrospective cohort (9 [7; 18] vs. 5 [4; 11] days; Z = −2.09; *p* = 0.0347).

**Figure 2:**
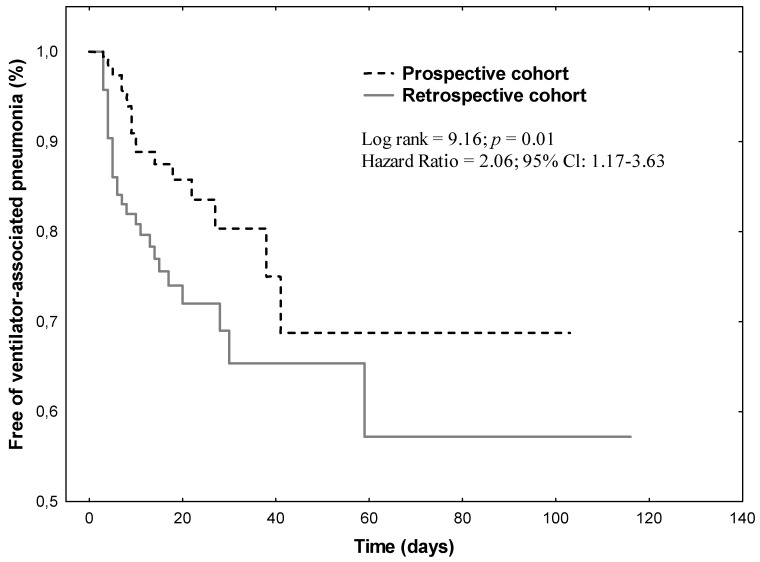
Kaplan–Meier curves of cumulative rates of patients remaining free of ventilator-associated pneumonia in two cohorts (the risk of ventilator-associated pneumonia is 2.06 times higher in the retrospective cohort than in the prospective cohort).

The cumulative rates of patients remaining VAP-free in the two cohorts using the Kaplan–Meier curve showed that the rate of VAP-free patients in the prospective cohort was higher than in the retrospective cohort (*p* = 0.01) (Figure 2).

### 3.4. Ventilator Days and Ventilator-Free Days

There was no significant difference in the median number of days of mechanical ventilation (8 [3; 21] vs. 8 [3; 15]; Z = 1.36; *p* = 0.17) and ventilator-free days (1 [0; 4] vs. 1 [0; 2]; Z = −0.17; *p* = 0.85) between the cohorts. Patients who developed VAP received mechanical ventilation for a median duration of 11 days, while the median of ventilator-free days was 5.5.

The VAP rate per 100 days of mechanical ventilation was not significantly different between the prospective and the retrospective cohorts (0.78; 95% CI: 0.48 to 1.2 vs. 1.12; 95% CI: 0.79–1.56; *p* = 0.21)

### 3.5. ICU Length of Stay

Medians ICU stays were similar in the prospective and retrospective cohorts (9 [4; 21] vs. 10 [4; 25] days; *p* = 0.43). Over 60% of the patients were hospitalized for more than 5 days, regardless of their group allocation (64.1% vs. 60.1%; *p* = 0.42).

There was no significant difference in median ICU stay between patients who had acquired VAP in the prospective and retrospective cohorts (22 [18; 63] days vs. 52 [19; 77] days; Z = 1.07; *p* = 0.28).

### 3.6. Pathogens

The types of pathogens in the culture of the lower respiratory secretions of patients in the prospective and retrospective cohorts are presented in Table 3. Multidrug-resistant bacteria were found in 10.1% and 12.7% of patients (*p* > 0.05). The microbiological findings of endotracheal aspirate included mainly gram-negative bacteria, Enterobacteriaceae species being the most common. In the retrospective cohort, other Enterobacteriaceae, Methicillin-susceptible Staphylococcus aureus and Staphylococcus epidermidis, were found more often (*p* < 0.05).

### 3.7. Mortality

Mortality during the first 28 days of hospitalization (*n* = 87; 43.9% vs. *n* = 61; 35.3%; *p* = 0.09) and the entire stay at the ICU (*n* = 96; 48.5% vs. *n* = 70; 40.5%; *p* = 0.12) was similar in the prospective and retrospective cohorts.

Among the patients diagnosed with VAP, in the prospective cohort, 6 patients died during the first 28 days of hospitalization and 2 patients died during the further stay at the ICU (42.1%). In the retrospective cohort, the number of deaths among patients with VAP was 8 (24.2%), all cases being within the first 28 days of hospitalization.

Among the group of deceased with VAP (*n* = 16), the presence of the MDR pathogen in the lower respiratory tract secretion was noted in 50% of cases (5 in the prospective cohort, 3 in the retrospective cohort).

## 4. Discussion

The results of the study show that the supposed modified VAP bundle (continuous pressure control in the artificial airway cuff with continuous automatic subglottic secretion suction) reduced the total incidence of VAP and early VAP, as well as extended the time to VAP in mechanically ventilated patients.

In the study, the authors used endotracheal/tracheotomy tubes with a taper-shaped cuff and the subglottic suction system. In in vitro studies [19,20] and in an ex vivo animal study [26], taper-shaped cuffs provided better sealing properties than cylindrical or spherical ones. Therefore, the use of an artificial airway with a taper-shaped cuff should be effective in the prevention of VAP by limiting the micro-aspiration of infected secretions to the lower respiratory tract. Unfortunately, it seems that the results of the meta-analysis conducted by Huang et al. [21] and a systematic review and meta-analysis by Saito et al. [27] support the thesis that neither the shape of cuffs (spherical, cylindrical, taper-shaped), nor the type of material they consist of (polyvinyl chloride, polyurethane), have an influence on the incidence of VAP. However, high-quality testing is needed to clearly assess the importance of the material and the shape of the tube cuff. Moreover, cuff modifications alone may not be sufficient to prevent VAP, therefore, they must be complemented by other interventions.

One of the important elements of VAP prevention is to ensure constant pressure in the artificial airway cuff, i.e., within the range of 20–30 mmHg. Maintaining constant pressure not only reduces the risk of micro-aspiration, but also prevents harmful consequences of the cuff slipping over the tracheal wall. Based on an in vitro study designed by Li Bassi et al. [28], Huang et al. [21] suggested the need for continuous pressure control in tapered cuffs. Li Bassi et al. [28] showed that tapered cuffs have a smaller contact surface with the tracheal wall than those with a spherical or cylindrical shape, which according to Huang et al. [21] may, due to pressure fluctuations, lead to the sliding of the cuff along the tracheal wall. In our study, as in the research conducted by Lorente et al. [29], maintaining constant pressure in the artificial airway cuff at a minimum of 25 mmHg was possible thanks to the use of an electronic device. The aforementioned researchers achieved an approximately 11% reduction in the incidence of VAP in the group of patients subjected to the continuous pressure control compared to the group with the intermittent control of cuff pressure using a manometer. The researchers concluded that the use of the continuous pressure control system and/or endotracheal tube with a lumen for SSS may prevent VAP [29] and reduce health care costs [30]. In our study, the combined use of these methods resulted in a reduction of the incidence of VAP by about 10%, however, we applied subglottic secretion suction using a continuous method, not intermittently during 1 h periods with a 10 mL syringe, as in the cited study.

The validity of using subglottic secretion suction was confirmed by the meta-analysis conducted by Mao et al. [17]. The procedure, both continuous and intermittent, depending on the quality of the study, reduced the probability of VAP (from 45% to 46%) and significantly diminished the incidence of early-onset VAP as well as delayed the time to VAP. Similar results were obtained in our study. The modified VAP bundle reduced the risk of total VAP by 50% and early VAP by as much as 81%. However, neither in our study nor in the cited meta-analysis, reduction in the incidence of late VAP was noted, as was the case in the study conducted by Vijai et al. [31] and Mahmoodpoor et al. [32] using the intermittent suction technique. However, due to the limited amount of scientific evidence, it is difficult to clearly determine which method is superior, intermittent or continuous suction. Serious complications such as tracheal damage, for instance, have only been observed in animals subjected to subglottic secretion suction [33]. This complication was not found in our study.

In our study, there was no effect of the modification of the VAP prevention bundle on the number of days of mechanical ventilation, mechanical-free days, hospitalization in the ICU and mortality, similar to the randomized study conducted by Mahmoodpoor et al. [32]. Although the incidence of VAP was reduced in our study and the research conducted by the cited authors, the mortality rate remained unchanged, suggesting that other confounding factors may have influenced the results of both studies.

### 4.1. Strengths and Limitations

The study was conducted in two large-scale cohorts, i.e., prospective and retrospective. The authors did not apply a propensity score matching between the two cohorts of patients, which could be a source of bias in estimating treatment effects. The non-simultaneous use of various VAP prevention bundles could be a confounding factor due to the subconscious over accuracy in the bundle implementation by nurses in the prospective cohort. It may also have made it difficult to analyze the results of lower airway secretion cultures in the two cohorts. In addition, in the retrospective cohort, endotracheal and tracheostomy tube cuffs made of polyvinyl chloride were respectively spherical and cylindrical. In contrast, in the prospective cohort, taper-shaped cuffs were made of polyurethane only in the case of tracheostomy tubes. As recent reports indicate no influence of the cuff shape and material on the incidence of VAP, we concluded that these elements of anesthesia equipment would not introduce an error into our analysis. The study results also cannot be generalized to all patients hospitalized in intensive care units, as it is a single-center study. There is also no data on the costs of the intervention.

### 4.2. Practical Implications of the Study

The study does not provide answers to the questions whether and to what extent the shape of the artificial airway cuff contributed to the reduction of VAP. It also does not indicate which of the methods of VAP prevention (automatic constant pressure control in the cuff or automatic constant subglottic secretion suction) influenced the final effectiveness. All these elements should be considered together.

The positive effects of the modifications of the VAP prevention bundle were the reduction of the total number of VAP, early VAP, and the extension of the period until the first symptoms of VAP. The results of the study served as the basis to introduce changes to the routine-preventive measures applied at our ICU. The study results support the hypothesis that implementing a comprehensive evidence-based bundle is effective in reducing the incidence of VAP [15]. An additional desired effect of the modification in the VAP prevention was to facilitate the work of nursing staff. Relieving the nurses from the necessity to perform multiple daily manual activities related to subglottic secretion suction and control of the pressure in the artificial airway cuff made it possible for them to perform a greater number of other nursing and therapeutic procedures. The results of the study conducted by Aeppli et al. [34] also confirm the usefulness of constant pressure control in the cuff. They showed that every maneuver of pressure control in cuffs by nurses using manual manometers may lead to under-inflation of the cuffs, and thus favor micro-aspiration of the secretion accumulated above the cuff into the bronchial tree.

Adherence to the rules of VAP prevention has been identified as one of the important factors in reducing the incidence of VAP [15]. Therefore, before the implementation of the modified VAP prevention bundle, the entire ICU medical and nursing team was trained, and the quality of activities related to VAP prevention was monitored on a daily basis. This allowed for a thorough understanding of the VAP problem by all employees of the ward, which could contribute to improving the quality of care for patients.

## 5. Conclusions

In this single centered retrospective-prospective study on unmatched cohorts of patients, we found that continuous pressure control in the artificial airway tapered cuff with continuous automatic subglottic secretion suction from above the cuff can reduce the total incidence of VAP and early VAP, as well as increase the time to VAP in mechanically ventilated ICU patients.

## Figures and Tables

**Table 1:** Chest Echocardiography and Procalcitonin Pulmonary Infection Score (CEPPIS) used in the diagnosis of VAP in the prospective and retrospective cohort (CEPPIS > 5 = VAP).

Parameter	CEPPIS
Temperature (°C)	
≥38.5 and <38.9	1
≥39 and <36	2
Prokalcitonin (ng/mL)	
≥0.5 and <1	1
≥1	2
Purulent tracheal secretions	2
Positive infiltrates on chest echograph (sub-pleural echo-poor region or more with tissue-like echo texture)	2
Positive endotracheal aspirate (>10^4^ colony-formingunits/mL)	2
Oxygenation PaO_2_/FiO_2_ ≤ 240 and absence of ARDS	2

VAP—ventilator-associated pneumonia; ARDS—acute respiratory distress syndrome.

**Table 2:** Patient characteristics.

Variables	Prospective (*n* = 198)	Retrospective (*n* = 173)	*p* *Value*
Age (years)	64.0 [50.0; 73.0]	68.0 [56.0; 76.0]	0.0638
Body mass index (kg/m^2^)	26.0 [23.0; 29.0]	26.0 [24.0; 29.4]	0.4151
APACHE II scores	23.0 [18.0; 30.0]	24.0 [20.0; 28.0]	0.5395
Sex			
Female	68 (34.3)	55 (31.8)	0.6025
Male	130 (65.7)	118 (68.2)	
Admission category			
Neurosurgical	88 (44.5)	92 (53.2)	0.0930
Cardiovascular	38 (19.2)	39 (22.5)	0.4271
General surgical	44 (22.2)	19 (11.0)	0.004
Non-cardiac internal medicine	28 (14.4)	23 (13.3)	0.8132
Chronic heart failure	35 (17.7)	38 (22.0)	0.2999
Chronic renal failure	17 (8.6)	18 (10.4)	0.5499
Chronic liver failure	8 (4.0)	1 (0.6)	0.03
Chronic obstructive pulmonary disease	22 (11.1)	18 (10.4)	0.8135
Gastric and duodenal ulcer	9 (4.5%)	7 (4.0)	0.8133
Diabetes mellitus	46 (23.2)	43 (24.9)	0.7149
Immunosuppression	12 (6.1)	5 (2.9)	0.1451
Smoking status	43 (21.7)	39 (22.5)	0.8482
Septic shock	40 (20.2)	30 (17.3)	0.4822
Tracheotomy	54 (27.3)	59 (34.1)	0.1537
Enteral nutrition	169 (85.4)	153 (88.4)	0.3811
Antibiotics prior to VAP	36 (18.2)	36 (20.8)	0.5232

Results presented as medians (upper and lower quartile) or numbers (percentages). In the prospective cohort, patients were hospitalized more often for general surgical reasons than in the retrospective cohort (*p* = 0.004). There were more cases of chronic liver failure in this cohort too (*p* = 0.03). There was no statistically significant difference between the other variables (*p* > 0.05).

**Table 3:** Microbiologic pathogens isolated from the lower respiratory secretions of patients in the prospective and retrospective cohorts.

Pathogens	Prospective (*n* = 198)	Retrospective (*n* = 173)	*p* *Value*
Multidrug-resistant bacteria	20 (10.1)	22 (12.7)	0.4276
Gram-positive bacteria			
Methicyllin-resistant Staphylococcus aureus	3 (1.5)	3 (1.7)	0.8675
Methicillin-susceptible Staphylococcus aureus	7 (3.5)	15 (8.7)	0.0367
Staphylococcus epidermidis	7 (3.5)	15 (8.7)	0.0367
Vancomycin-resistant enterococcus	0 (0.0)	2 (1.2)	0.1292
Streptococcus pneumoniae	6 (3.0)	10 (5.8)	0.1933
Gram-negative bacteria			
Acinetobacter baumanii	13 (6.6)	5 (2.9)	0.1002
Pseumonas aeruginosa	4 (2.0)	5 (2.9)	0.5869
Klebsiella pneumoniae (Enterobacteriaceae)	16 (8.1)	25 (14.4)	0.0509
Other Enterobacteriaceae	21 (10.6)	31 (17.9)	0.0429
Haemophilus influenzae	6 (3.0)	10 (5,8)	0.1933
Stenotrophomonas maltofila	7 (3.5)	11 (6.4)	0.2068
Candida albicans	4 (2.0)	0 (0.0)	0.0601

Results presented as numbers and percentages.

## Data Availability

A dataset will be made available upon request to the corresponding authors one year after the publication of this study. The request must include a statistical analysis plan.

## Abbreviations

ARDS	acute respiratory distress syndrome
CEPPIS	Chest Echocardiography and Procalcitonin Pulmonary Infection Score
Cl	confidence interval
ICU	intensive care units
OR	odds ratio
PEEP	positive end-expiratory pressure
RR	relative risk
SSS	subglottic secretion suction
VAP	ventilator-associated pneumonia
VT	tidal volume

## References

[1] Marshall J.C., Bosco L., Adhikari N.K., Bronwen Connolly B., Diaz J.V., Dorman T., Fowler R.A., Meyfroidt G., Nakagawa S., Pelosi P. (2017). What is an intensive care unit? A report of the task force of the World Federation of Societies of Intensive and Critical Care Medicine. J. Crit. Care.

[2] Papazian L., Klompas M., Luyt C.E. (2020). Ventilator-associated pneumonia in adults: A narrative review. Intensive Care Med..

[3] Torres A., Niederman M.S., Chastre J., Ewig S., Fernandez-Vandellos P., Hanberger H., Kollef M., Bassi G.L., Luna C.M., Martin-Loeches I. (2017). International ERS/ESICM/ESCMID/ALAT guidelines for the management of hospital-acquired pneumonia and ventilator-associated pneumonia: Guidelines for the management of hospital-acquired pneumonia (HAP)/ventilator-associated pneumonia (VAP) of the European Respiratory Society (ERS), European Society of Intensive Care Medicine (ESICM), European Society of Clinical Microbiology and Infectious Diseases (ESCMID) and Asociación Latinoamericana del Tórax (ALAT). Eur Respir. J..

[4] Ego A., Preiser J.C., Vincent J.L. (2015). Impact of diagnostic criteria on the incidence of ventilator-associated pneumonia. Chest.

[5] Steen J., Vansteelandt S., De Bus L., Depuydt P., Gadeyne B., Benoit D.D., Decruyenaere J. (2021). Attributable Mortality of Ventilator-associated Pneumonia. Replicating Findings, Revisiting Methods. Ann. Am. Thorac. Soc..

[6] Ding C., Zhang Y., Yang Z., Wang J., Jin A., Wang W., Chen R., Zhan S. (2017). Incidence, temporal trend and factors associated with ventilator-associated pneumonia in mainland China: A systematic review and meta-analysis. BMC Infect. Dis..

[7] Bonell A., Azarrafiy R., Huong V.T.L., Le Viet T., Phu V.D., Dat V.Q., Wertheim H., van Doorn H.R., Lewycka S., Nadjm B. (2019). A Systematic Review and Meta-analysis of Ventilator-associated Pneumonia in Adults in Asia: An Analysis of National Income Level on Incidence and Etiology. Clin. Infect. Dis..

[8] Wałaszek M., Różańska A., Wałaszek M.Z., Wójkowska-Mach J. (2018). Polish Society of Hospital Infections Team. Epidemiology of Ventilator-Associated Pneumonia, microbiological diagnostics and the length of antimicrobial treatment in the Polish Intensive Care Units in the years 2013–2015. BMC Infect. Dis..

[9] Duszynska W., Rosenthal V.D., Szczesny A., Zajaczkowska K., Fulek M., Tomaszewski J. (2020). Device associated -health care associated infections monitoring, prevention and cost assessment at intensive care unit of University Hospital in Poland (2015–2017). BMC Infect. Dis..

[10] Kharel S., Bist A., Mishra S.K. (2021). Ventilator-associated pneumonia among ICU patients in WHO Southeast Asian region: A systematic review. PLoS ONE.

[11] Isac C., Samson H.R., John A. (2021). Prevention of VAP: Endless evolving evidences-systematic literature review. Nurs. Forum.

[12] Phu V.D., Nadjm B., Duy N.H.A., Co D.X., Mai N.T.H., Trinh D.T., Campbell J., Khiem D.P., Quang T.N., Loan H.T. (2017). Ventilator-associated respiratory infection in a resource-restricted setting: Impact and etiology. J. Intensive Care.

[13] Pileggi C., Mascaro V., Bianco A., Nobile C.G.A., Pavia M. (2018). Ventilator Bundle and Its Effects on Mortality Among ICU Patients: A Meta-Analysis. Crit. Care Med..

[14] Alecrim R.X., Taminato M., Belasco A., Longo M.C.B., Kusahara D.M., Fram D. (2019). Strategies for preventing ventilator-associated pneumonia: An integrative review. Rev. Bras. Enferm..

[15] Álvarez-Lerma F., Sánchez García M. (2018). Task Force of Experts for Project “Zero VAP” in Spain. “The multimodal approach for VAP prevention”—Requirements for nationwide implementation. Ann. Transl. Med..

[16] Nseir S., Lorente L., Ferrer M., Rouzé A., Gonzalez O., Bassi G.L., Duhamel A., Torres A. (2015). Continuous control of tracheal cuff pressure for VAP prevention: A collaborative meta-analysis of individual participant data. Ann. Intensive Care.

[17] Mao Z., Gao L., Wang G., Liu C.H., Zhao Y., Gu W., Kang H., Zhou F. (2016). Subglottic secretion suction for preventing ventilator-associated pneumonia: An updated meta-analysis and trial sequential analysis. Crit. Care.

[18] Pozuelo-Carrascosa D.P., Herráiz-Adillo Á., Añón J.M., Martínez-Vizcaíno V., Cavero-Redondo I. (2020). Subglottic secretion drainage for preventing ventilator-associated pneumonia: An overview of systematic reviews and an updated meta-analysis. European Respiratory Review: An Official. J. Eur. Respir. Soc..

[19] Maguire S., Haury F., Jew K. (2015). An in vitro comparison of tracheostomy tube cuffs. Med. Devices.

[20] Khashaba S.A., Chaari A., Uddin F., Hamamah D., Ismail M., Tierney E., Casey W.F. (2019). Tapered-cuff versus cylindrical-cuff tracheal tube in preventing fluid leak: An in-vitro experimental study. Trends Anaesthesia Crit. Care.

[21] Huang W.M., Huang X.A., Du Y.P., Huang W.M., Huang X.A., Du Y.P., Li L.X., Wu F.F., Hong S.Q., Tang F.X. (2019). Tapered Cuff versus Conventional Cuff for Ventilator-Associated Pneumonia in Ventilated Patients: A Meta-Analysis of Randomized Controlled Trials. Can. Respir. J..

[22] Maertens B., Blot S. (2019). Comment on “Tapered Cuff versus Conventional Cuff for Ventilator-Associated Pneumonia in Ventilated Patients: A Meta-Analysis of Randomized Controlled Trials”. Can. Respir. J..

[23] Vandenbroucke J.P., von Elm E., Altman D.G., Vandenbroucke J.P., von Elm E., Altman D.G., Gøtzsche P.C., Mulrow C.D., Pocock S.J., Poole C.H. (2014). Strengthening the Reporting of Observational Studies in Epidemiology (STROBE): Explanation and elaboration. Int. J. Surg..

[24] Knaus W.A., Draper E.A., Wagner D.P., Zimmerman J.E. (1985). APACHE II: A severity of disease classification system. Crit. Care Med..

[25] Zhao T., Wu X., Zhang Q., Li C., Worthington H.V., Hua F. (2020). Oral hygiene care for critically ill patients to prevent ventilator-associated pneumonia. Cochrane Database Syst. Rev..

[26] Monsel A., Le Corre M., Deransy R., Brisson H., Arbelot C.H., Lu Q., Golmard J.L., Langeron O., Rouby J.J. (2017). Modification of Tracheal Cuff Shape and Continuous Cuff Pressure Control to Prevent Microaspiration in an Ex Vivo Pig Tracheal Two-Lung Model. Crit. Care Med..

[27] Saito M., Maruyama K., Mihara T., Hoshijima H., Hirabayashi G., Andoh T. (2021). Comparison of polyurethane tracheal tube cuffs and conventional polyvinyl chloride tube cuff for prevention of ventilator-associated pneumonia: A systematic review with meta-analysis. Medicine.

[28] Li Bassi G., Ranzani O.T., Marti J.D., Giunta V., Luque N., Isetta V., Ferrer M., Farre T., Pimentel G.L., Torres A. (2013). An in vitro study to assess determinant features associated with fluid sealing in the design of endotracheal tube cuffs and exerted tracheal pressures. Crit. Care Med..

[29] Lorente L., Lecuona M., Jiménez A., Lorenzo L., Roca I., Cabrera J., Llanos C., Mora M.L. (2014). Continuous endotracheal tube cuff pressure control system protects against ventilator-associated pneumonia. Crit. Care.

[30] Lorente L., Lecuona M., Jiménez A., Cabrera J., Mora M.L. (2014). Subglottic secretion drainage and continuous control of cuff pressure used together save health care costs. Am. J. Infect. Control.

[31] Vijai M.N., Ravi P.R., Setlur R., Vardhan H. (2016). Efficacy of intermittent sub-glottic suctioning in prevention of ventilator-associated pneumonia- A preliminary study of 100 patients. Indian J. Anaesth..

[32] Mahmoodpoor A., Hamishehkar H., Hamidi M., Shadvar K., Sanaie S., Golzari S.E., Khan A.H., Nader N.D. (2017). A prospective randomized trial of tapered-cuff endotracheal tubes with intermittent subglottic suctioning in preventing ventilator-associated pneumonia in critically ill patients. J. Crit. Care.

[33] Berra L., De Marchi L., Panigada M., Yu Z.X., Baccarelli A., Kolobow T. (2004). Evaluation of continuous aspiration of subglottic secretion in an in vivo study. Crit. Care Med..

[34] Aeppli N., Lindauer B., Steurer M.P., Weiss M., Dullenkopf A. (2019). Endotracheal tube cuff pressure changes during manual cuff pressure control manoeuvres: An in-vitro assessment. Acta Anaesthesiol. Scand..

